# Supplementary material for: Tailoring Tofacitinib Oral Therapy in Rheumatoid Arthritis: The TuTOR App
Source: Int J Environ Res Public Health. 2022 Apr 28;19(9):5379. doi: 10.3390/ijerph19095379 (PMC9102425; doi:10.3390/ijerph19095379)
Supplement: Supplementary file 1 [file ijerph-19-05379-s001.zip › Supplementary.pdf]

Supplementary Materials:

Table S1. Baseline educational level, affinity with technology and personal information of the patients enrolled in the study, who completed follow-up. *RA—RheumatoidArthritis.*

	**Patients with RA (N = 17)**
**EDUCATIONAL LEVEL**	
Elementary school (n; %)	3; 18
Middle school (n; %)	5; 29
High school (n; %)	7; 41
University (n; %)	2; 12
**USE OF PERSONAL COMPUTER**	
At work (n; %)	9; 53
Confidence of Use on a scale from 1–100 (mean ± S.D.)	70 ± 26,5
**USE OF TABLET**	
At work (n; %)	2; 12
Confidence of Use on a scale from 1–100 (mean ± S.D.)	75 ± 23,8
**USE OF SMARTPHONE**	
At work (n; %)	4; 23.5
Confidence of Use on a scale from 1–100 (mean ± S.D.)	93,3 ± 11,5
**LIVING SITUATION**	
Urban area (n; %)	13; 76
Suburban area (n; %)	4; 24
**MARITAL STATUS**	
Conjugated (n; %)	13; 76
Separated (n; %)	3; 18
**WORK STATUS**	
Currently working (n; %)	11; 65
Currently retired (n; %)	5; 29
